# Dual-Task Effects During a Motor-Cognitive Task in Parkinson’s Disease: Patterns of Prioritization and the Influence of Cognitive Status

**DOI:** 10.1177/1545968321999053

**Published:** 2021-03-10

**Authors:** Hanna Johansson, Urban Ekman, Linda Rennie, Daniel S. Peterson, Breiffni Leavy, Erika Franzén

**Affiliations:** 1 Karolinska Institutet, Stockholm, Sweden; 2 Karolinska University Hospital, Stockholm, Sweden; 3 Sunnaas Rehabilitation Hospital, Nesodden, Norway; 4 Arizona State University, Phoenix, AZ, USA; 5 Phoenix Veterans Affairs Health Care System, Phoenix, AZ, USA; 6 Stockholm Sjukhem Foundation, Stockholm, Sweden

**Keywords:** Parkinson disease, gait, multitasking behavior, cognition

## Abstract

People with Parkinson’s disease (PD) experience greater difficulties during dual task (DT) walking compared to healthy controls, but factors explaining the variance in DT costs remain largely unknown. Additionally, as cognitive impairments are common in PD it is important to understand whether cognitive status influences the strategies used during DT paradigms. The study aimed to (1) explore DT costs on gait and cognition during DT walking, (2) investigate factors associated with DT costs, and (3) to investigate to what extent patterns of DT costs and prioritization differed according to cognitive status. A total of 93 people with Parkinson’s disease were examined when walking in single and DT conditions. Information regarding demographics, PD severity, mobility, and cognitive and affective symptoms was collected, and an extensive neuropsychological test battery was used to classify whether participants had mild cognitive impairment (PD MCI) or not (PD non-MCI). Dual task costs were observed across all gait domains except asymmetry. Cognitive status was associated with DT costs on both gait and cognition. Nonmotor experiences of daily living were further associated with DT cost on cognition, and TUG-cog associated with DT cost on gait. People with PD MCI had larger DT costs on gait than PD non-MCI. Strategies differed according to cognitive status, whereby PD MCI used a posture-second strategy, and PD non-MCI used a posture-first strategy. Once verified in future studies, these results can inform clinicians and researchers when tailoring DT training paradigms to the specific characteristics of people with PD.

## Introduction

Cognitive impairments are common in Parkinson’s disease (PD), and one of the more prominent features is dysfunction within the executive function domain.^1^ Executive dysfunction affects the ability to inhibit prepotent responses, to update working memory responses, and to perform mental set shifting.^2^ Executive function is also central when performing 2 tasks simultaneously, that is, dual tasking, and this area of PD research has gained considerable attention over the last decades. The proportion by which performance of a task is affected by the simultaneous performance of a second task can be referred to as a dual task effect (DT effect), where poorer performance is noted as a DT cost and improved performance as a DT benefit. It has been suggested that people with PD (PwPD) experience greater difficulties when performing DTs compared to healthy controls.^3^ A recent meta-analysis showed that adding a DT during walking has a moderate to large negative effect on gait speed in PwPD, regardless of single task gait speed or type of dual task.^4^ To what extent this is also reflected in other gait parameters is less evident. The DT effect has been suggested as a proxy measure for attention and automaticity,^5^ and establishing factors associated with DT effect on gait may help identify those most at risk during everyday DT activities and DT training. Investigations to date, however, are insufficient in explaining the variance of this important proxy measure.

Evidence for the DT effect on the secondary task is sparse in the literature, even though performance of a secondary cognitive task can be an even better predictor of motor impairment than gait performance during DT conditions.^6^ This paucity of evidence regarding the DT effect on the secondary task results in an inability to decipher how and to what extent one task has been prioritized over the other. Interpreting an individual’s process of prioritization during dual tasking may provide essential information that help us tailor our interventions. It has been suggested that during DT walking conditions, PwPD may use a hazardous posture-second strategy, whereby the secondary task is prioritized over safe walking.^7^ However, it has also been shown that in the PD population, those without cognitive impairment have the ability to shift focus and change to a posture-first strategy.^8^

Approximately 40% of people newly diagnosed with PD have already developed mild cognitive impairment (PD MCI).^9,10^ Imaging studies reveal that PD MCI compared to PD non-MCI have a more advanced cortical degeneration,^11^ and task-evoked underrecruited activity in the bilateral anterior cingulate cortex and the right dorsal caudate nucleus suggesting a link between cognitive impairment and frontostriatal dysfunction.^12^ Previous studies indicate that PwPD MCI have an altered gait pattern, compared to those without MCI during usual walking, such as shorter stride and step length.^13^ However, these differences in gait pattern do not seem as apparent during DT conditions, nor are they reflected by significant differences in DT costs on gait.^14,15^ If we are to design and safely implement DT training paradigms for all PwPD, not only those without cognitive impairment, we require a better understanding of whether PwPD MCI differ from those without MCI with regard to DT costs and task prioritization.

The primary aim of this study was to explore DT effects during simultaneous performance of a motor task (gait) and a cognitive task (auditory Stroop task) in people with mild to moderate PD. The study also had secondary aims. The first was to investigate to which extent factors related to demographics, PD severity, mobility, cognitive status, and affective symptoms were associated with DT effects on gait and cognitive performance, respectively. Based on previous studies we hypothesized that motor function and cognitive status would be associated with the DT effect on gait speed.^5,16^ Last, we also aimed to explore any potential differences in patterns of DT effect and prioritization in PD MCI and PD non-MCI.

## Materials and Methods

### Design and Setting

This study used a cross-sectional design based on baseline data from the EXercise in Parkinson’s disease and Neuroplasticity Trial (EXPANd; registered at clinicaltrials.gov NCT03213873).^17^ Data collection commenced in January 2018 and concluded in August 2019. Assessments were performed in a university setting.

### Participants

Participants were recruited through advertisements in the Swedish Parkinson Association and in newspapers. Participants were eligible for inclusion if they (1) had a diagnosis of Idiopathic PD, (2) were Hoehn and Yahr stage 2 to 3,^18^ (3) were ≥60 years of age, (4) scored ≥21 on Montreal Cognitive Assessment (MoCA), and (5) were able to ambulate indoors without a mobility aid. Participants were excluded if they had any other disorder that substantially influenced gait performance.

### Procedures and Outcomes

All participants were assessed in their ON stage of levodopa medication. Assessment of descriptors and DT gait, and neuropsychological assessment were performed on 2 separate days in order to minimize fatigue.

#### Descriptors

We collected demographic information on age, height, weight, years of education, disease duration, and medication intake. Balance and motor function were assessed with the Mini Balance Evaluation Systems Test (Mini-BESTest)^19^ and the Movement Disorder Society Unified Parkinson’s Disease Rating Scale (MDS-UPDRS), part III (motor examination), respectively.^20^ For the purpose of this study, the last item on the Mini-BESTest, Timed Up and Go Cognitive (TUG-cog), was also used as a separate outcome. Whereas TUG-cog within the Mini-BESTest is scored between 0 and 2, when used as a separate variable in this study the number of seconds to complete the test was utilized as the outcome. Participants also answered self-report questionnaires pertaining to their balance and gait ability using Activities-specific Balance Confidence scale^21^ and Walk-12,^22^ nonmotor and motor experiences of daily living using MDS-UPDRS parts I and II,^20^ and quality of life and health status using EuroQol 5 Dimensions,^23^ Parkinson’s Disease Questionnaire–39 (PDQ-39),^24^ and Hospital Anxiety and Depression scale.^25^ Global cognition was assessed using MoCA as part of eligibility screening.^26^

#### Classification of Cognitive Impairment

A neuropsychological test battery was performed comprising the following domains: executive function, attention/working memory, episodic memory, and visuospatial functions. See Table 1 for detailed information on which tests were included. Participants were classified as having MCI (PD-MCI) or not (PD non-MCI) according to the Movement Disorder Society task force level II category,^27^ which requires 2 tests within each of the 5 cognitive domains. We used trial IV from Color-Word Interference Test and trial II from verbal fluency (semantic fluency) for the executive function domain, digit span (total score) and trial IV of Trail Making Test for attention/working memory domain, and delayed recall from both RAVLT and BVMT-R for episodic memory domain. For the visuospatial domain we used the Copy condition from BVMT-R and the wire cube subtest from MoCA. For the purpose of this classification, the initial scoring of the wire cube (0-1) was rescored according to the 0-2 scoring in Addenbrooke cognitive examination,^28^ and normative comparison values used by Charernboon et al.^29^ Normative values for BVMT–copy trial were ascertained from Romero et al.^30^ For the language domain, the Naming and Sentences subtests from MoCA were used, and normative values from Borland et al were adopted.^31^

**Table 1.** Overview of the Neuropsychological Test Battery.

Domain	Test
Executive function	The Color-Word Interference Test (CWIT)^a^
	Verbal Fluency^b^
Attention and working memory	Digit Span^c^
	Trail Making Test (TMT), Trials I-IV^d^
Episodic memory	Rey Auditory Verbal Learning Test (RAVLT)^e^
	Brief Visuospatial Memory Test–Revised (BVMT-R)
Visuospatial functions	Copy condition from BVMT-R

a CWIT, from Delis Kaplan Executive Function System (D-KEFS).

b Verbal fluency, from D-KEFS.

c Digit span from Wechsler Adult Intelligence Scale (WAIS)–fourth edition, Swedish version.

d TMT from D-KEFS.

e RAVLT, version 1.

In each of these 10 test measures participants were given a score between 1 and 4 depending on their performance in relation to the normative mean values. Cutoffs used for each score were the following: 1 = ≤1 standard deviation (SD), 2 = 1.01 to 1.49 SD, 3 = 1.50 to 1.99 SD, and 4 = ≥2 SD. If a participant scored 3 or 4 on ≥2 tests they were classified as PD MCI. If a participant scored 1 on all tests or had a maximum of one test scored as 2, 3, or 4, they were classified as PD non-MCI. If a participant scored 2 on ≥2 tests or scored 2 on one test and 3 or 4 on one test, they were instead put in an intermediate group and excluded from analysis for the secondary aims.

#### Dual Task Gait Assessment

An electronic walkway system (GAITRite, active zone: 8.3 m, CIR Systems, Inc) was used to measure temporal and spatial gait parameters during single and DT conditions. Acceleration and deceleration distances of 3 m on each side of the mat were used to ensure steady state walking.^32^ During both conditions participants walked back and forth on the walkway a total of 8 trials, whereof the first two were considered practice runs and excluded from analysis. Participants always started with the single gait task and were instructed to walk at self-selected usual speed.

A cognitive task addressing executive functions such as set shifting and inhibition was then introduced. The auditory Stroop task was selected as it has been previously proven valid^33^ and reliable (gait speed intraclass correlation coefficient [ICC] .91, and reaction time ICC .82)^34^ during DT gait assessments in PD. The auditory Stroop task has been proven feasible to perform within the current context,^35^ and also allows for the analysis of multiple performance outcomes (such as reaction times, intraindividual variability of reaction times, as well as accuracy). Participants were presented with the Swedish words for “high” and “low” in congruent and incongruent high and low tones via wireless headphones (RazerTM ManO’War). They were instructed to respond verbally to the corresponding tone, irrespective of which word was presented, as fast as possible. Variable interstimulus intervals of 1.5 to 2.0 seconds were used in order to control for cueing effects. Participants were given 2 standardized practice trials (or more if needed for task comprehension) in a seated position, and 2 practice trials of walking while performing the auditory Stroop task (DT gait). The number of practice trials ranged between 2 and 4 (mean 2.24, SD 0.54). After the practice trials, a randomization process (computerized random sequence generator; http://www.randomization.com) decided whether participants were to start with the cognitive single task (auditory Stroop in seated position) or gait DT (walking while performing the auditory Stroop task). During DT gait, participants were instructed to pay equal attention to both tasks, and to start walking after they had responded to the first stimulus. The first stimulus of each walking trial was therefore excluded from the analyses.

#### Dual Task Data Analysis

According to the PD-gait model proposed by Lord et al,^36^ 16 spatiotemporal gait variables associated with pace, rhythm, variability, asymmetry, and postural control domains of PD-gait were determined as follows based on at least 40 steps. Mean values for step velocity, step length, step time, swing time, stance time, and step width were calculated. Gait asymmetry measures included swing time, step time, stance time, and step length and were calculated as the absolute difference between left leg and right leg. Gait variability included step velocity, step length, step width, step time, stance time, and swing time and was calculated in Excel (Excel, Microsoft) as described by Galna et al^37^ where the combined standard deviation (SD) of left and right steps was determined by taking the square root of the within-subject variance of the left and right steps as follows:

$$SD_{\mathrm{Left}\;\&\;\mathrm{Right}}=\sqrt{\frac{\left({\mathrm{Variance}}_{\mathrm{Left}\;\mathrm{Steps}}+{\mathrm{Variance}}_{\mathrm{Right}\;\mathrm{Steps}}\right)}{2}}$$

Absolute differences between single and DT performance were calculated as single task minus DT.

The DT effect was calculated as described by Kelly et al.^38^ For outcomes where a higher value indicated improvement/better performance the following equation was used:

$$DTE\left(\%\right)=\frac{\mathrm{Dual}\;\mathrm{task-Single}\;\mathrm{task}}{\mathrm{Single}\;\mathrm{task}}\;\times 100$$

For values where a lower value indicated improvement/better performance, a negative sign was inserted in the equation, thereby resulting in all negative DT effect values indicating a DT cost, and all positive DT effect values indicating a DT benefit.

Verbal responses to the auditory Stroop task were recorded using Audacity version 2.1.3,^39^ and analyzed using MATLAB (R2017b).^40^ Reaction times (RTs) were calculated as the time from beginning of the stimulus to beginning of the response. Mean RTs were calculated as the mean of all answers, irrespective of whether they were correct or incorrect, in each condition. The mean standard deviation of RT (SDRT) was used as a measure of intraindividual variability. Accuracy ratios were calculated for each condition. Nonresponses counted as incorrect answers. Dual task effects on accuracy and reaction times were calculated in the same manner as for gait. An overall DT effect on cognition was used for analysis of secondary aims and was calculated as follows:

$$\mathrm{DT}\;\mathrm{effect}\;\mathrm{on}\;\mathrm{cognition}=\frac{\begin{array}{l} \mathrm{DT}\;\mathrm{effect}\;\mathrm{on}\;\mathrm{reaction}\;\mathrm{time}\\ +\mathrm{DT}\;\mathrm{effect}\;\mathrm{on}\;\mathrm{accuracy} \end{array}}{2}$$

The ratio for congruent and incongruent Stroop stimuli were similar in both single and DT conditions. Participants were excluded from analysis if they had an accuracy score of <60% on the auditory Stroop task in DT condition as we wanted to be as certain as possible that they had focused on both tasks and not disregarded the cognitive task. Thus, this threshold was used to exclude answers by chance (ie, 50%), and to ensure that participants actually performed dual-task processing.

### Statistical Analysis

Data were analyzed using IBM SPSS Statistics for Mac version 26 (IBM Corp). Normality was assessed with kurtosis and skewness values, Shapiro-Wilks values, and by visual inspection of QQ-plots and histograms. For normally distributed data, mean and SDs are provided, and for nonnormally distributed data median and interquartile range are provided. Due to multiple nonnormally distributed gait parameters, all gait variables but gait speed and step length are presented in median and interquartile range. To assess whether there were significant differences between single and DT performance, Wilcoxons signed rank test was used.

For the secondary aims, linear regression analysis was used to identify factors associated with DT effect on primary gait and cognition outcomes (gait speed, step time variability, and cognition, that is, the ratio of the DT effect on reaction time added to the DT effect on accuracy). Gait speed was selected as this is the most commonly reported as well as clinically used gait variable. Step time variability was selected as an additional and more exploratory gait parameter of interest, because it has previously been used as a surrogate marker for both gait automaticity^41^ and fall risk.^42^ Finally, cognition was chosen as we wanted a variable that incorporated both reaction time and accuracy. Potential independent variables were selected based on clinical reasoning and/or correlation (*P* < .2) in univariate linear regression analysis between descriptive variables and the primary DT effect variables. Cutoff for multicollinearity was set at 0.6.

Extreme outliers (>3 SD from mean) in dependent variables were removed when they had an undue influence on the choice of independent variables, that is, when their inclusion altered which variables were correlated with the outcome variable to a large extent.

After independent variables had been selected, they were entered into the multiple regression models using backward selection. The first 2 models with DT effect on gait speed and step time variability as dependent variables respectively included the following independent variables: cognitive status (PD MCI/PD non-MCI), MDS-UPDRS II, Mini-BESTest total, and TUG-cog. The third model that had DT effect on cognition as the dependent variable included the following independent variables: cognitive status, education, years since diagnosis, MDS-UPDRS I, and TUG-cog. Multivariate outliers were identified as those with a Cooks distance of >4/(*n* − *k* − 1) and were controlled for incorrect values.^43^ Analyses were rerun without the multivariate outliers and coefficients in both models were compared to see how much influence the outliers had. Multivariate outliers were inspected but deemed to be correct. Therefore, a decision was made not to remove the multivariate outliers as they were a true part of the sample.

Prioritization was calculated by subtracting the DT effect on gait variables speed and step time variability from the DT effect on cognition (DT effect on cognition − DT effect on gait speed, and DT effect on cognition − DT effect on step time variability), whereby a negative value indicated gait task prioritization and a positive value indicated cognitive task prioritization. A single samples *t* test was run on the prioritization variable to determine if the mean was significantly different from zero. Between-group differences of the MCI group and the non-MCI group regarding DT effect on cognition, gait speed, and step time variability, as well as task prioritization was assessed using univariate linear regression. All models were also rerun and adjusted for sex and age. A *P* value <.05 was considered statistically significant for all analyses.

## Results

Baseline characteristics of included participants are presented in Table 2. A total of 117 participants were assessed for eligibility, whereof 96 were eligible for inclusion. Three of these participants had an accuracy score of <60% during DT walking and were therefore excluded from analysis. The mean age of the remaining 93 participants was 71.0 years, and approximately one third were female (n = 34). A total of 26 participants were classified as having PD MCI, 39 as PD non-MCI. and 24 as being intermediate between PD MCI and PD non-MCI.

**Table 2.** Descriptive Characteristics of Included Participants.

Characteristic, mean (SD) unless otherwise stated	N = 93
Sex, female, n (%)	34 (36.6)
Age (years)	71.0 (6.1)
Body mass index (kg/m^2^)	25.3 (3.5)
Education (years)	14.8 (3.0)
Years with diagnosis	5.2 (4.5)
Levodopa equivalent dose (mg/day)	554.1 (331.3)
Hoehn & Yahr	
2, n (%)	71 (76.3)
3, n (%)	22 (23.7)
People who fell in previous 6 months, n (%)	29 (31.2)
Montreal Cognitive Assessment (0-30)	25.8 (2.4)
Cognitive status (4 missing)	
PD non-MCI, n (%)	39 (41.9)
PD MCI, n (%)	26 (28.0)
Intermediate^a^, n (%)	24 (25.8)
Activities-specific Balance Confidence scale (0-100)	79.4 (16.2)
Walk-12 scale (0-46)	11.9 (8.4)
Mini Balance Evaluation Systems Test (0-28)	20.9 (3.5)
Timed Up and Go (seconds)	10.8 (2.7)
MDS-UPDRS, Part III, motor examination (0-132)	31.1 (11.2)
EuroQol Visual Analogue Scale (%)	72.7 (15.3)
PDQ-39, Summary Index (0-100)	20.7 (12.4)
HADS anxiety subscale (0-21)	4.2 (3.3)
HADS depression subscale (0-21)	3.2 (2.8)

Abbreviations: MDS-UPDRS, Movement Disorder Society–Unified Parkinson’s Disease Rating Scale; PDQ-39, Parkinson’s Disease Questionnaire–39; HADS, Hospital Anxiety and Depression Scale.

a Participants who could not with certainty be classified as either PD MCI or PD non-MCI.

### Dual Task Effects

**Figure 1.**
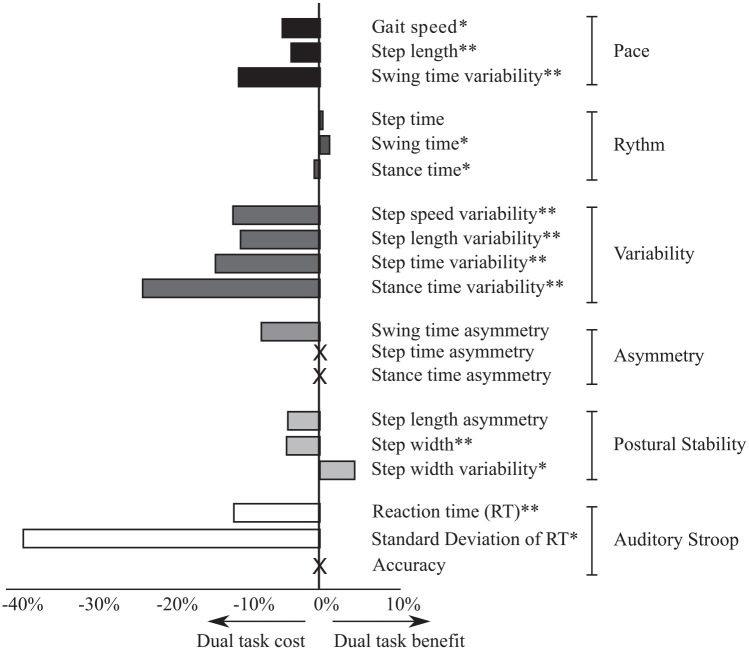
Overview of dual task effects on gait variables and the auditory Stroop task. *indicate those variables where the difference in performance between single and dual task was significant (**p < 0.001; *p < 0.05). A significant dual task cost means a decline in performance from dual task compared to single task.

Single and DT values, as well as DT effects for all gait parameters, and cognitive task outcomes are presented in Table 3. All gait variables within the pace domain and the variability domain showed DT cost, with a significant change from single to DT performance. Within the postural control domain, step width was wider from single to DT, with a DT cost of 4.2%. No changes were observed within the asymmetry domain. With regard to the cognitive task, RTs were significantly longer during DT compared to single task, whereas no difference in accuracy was observed. The intraindividual variability in reaction times (SDRT) also increased significantly during dual tasking, with a DT cost of 39.2%. All DT effects are compiled in Figure 1 for a schematic overview.

**Table 3.** Dual Task Effects for All Gait Parameters and Cognitive Outcomes^a^.

Domain	Variable	Single task	Dual task	*Z* value	*P* value	Dual task effect (%)
Pace	Gait speed (m/s), mean (SD)	1.19 (0.19)	1.13 (0.27)	−3.26	.001	−4.94 (11.91)
	Step length (m), mean (SD)	0.65 (0.09)	0.62 (0.09)	−4.84	<.001	−3.77 (7.09)
	Swing time variability (ms)	14.28 (6.66)	16.04 (8.90)	−4.57	<.001	−10.72 (32.47)
Rhythm	Step time (ms)	544.50 (43.00)	548.00 (58.50)	−1.31	.189	0.38 (7.2)
	Swing time (ms)	392.50 (35.50)	385.50 (43.00)	−2.10	.037	1.27 (4.27)
	Stance time (ms)	694.50 (71.75)	705.50 (98.25)	−2.53	.011	−0.75 (9.61)
Variability	Step speed variability (m/s)	0.04 (0.02)	0.05 (0.02)	−3.47	.001	−11.49 (40.20)
	Step length variability (m)	0.02 (0.01)	0.02 (0.01)	−3.84	<.001	−10.49 (42.12)
	Step time variability (ms)	14.38 (7.18)	16.53 (10.62)	−4.24	<.001	−13.81 (42.38)
	Stance time variability (ms)	16.49 (6.90)	20.24 (13.19)	−4.91	<.001	−23.42 (56.17)
Asymmetry	Swing time asymmetry (ms)	9.00 (11.50)	10.00 (10.00)	−0.65	.518	−7.74 (130.24)
	Step time asymmetry (ms)	12.00 (14.50)	13.00 (16.00)	−0.83	.404	0.00 (116.12)
	Stance time asymmetry (ms)	9.00 (10.50)	10.00 (12.50)	−0.264	.792	0.00 (130.36)
Postural control	Step length asymmetry (m)	0.02 (0.03)	0.02 (0.02)	−1.45	.147	−4.24 (109.39)
	Step width (m)	0.08 (0.03)	0.09 (0.03)	−4.50	<.001	−4.41 (11.76)
	Step width variability (m)	0.02 (0.01)	0.02 (0.01)	−2.16	.031	4.59 (28.15)
Cognition	Reaction time (RT) (s)	0.99 (0.26)	1.10 (0.13)	−5.39	<.001	−11.37 (24.68)
	Standard deviation of RT	0.30 (0.27)	0.37 (0.09)	−2.02	.043	−39.19 (126.29)
	Accuracy (%)	100.00 (6.67)	96.55 (8.70)	−1.36	.174	0.00 (4.19)

a Gait speed and step length (single task, dual task, and dual task effect) presented as mean (SD). All other presented as median (interquartile range). Dual task effect is presented in negative or positive values where negative values indicates a dual-task cost, and positive values a dual-task benefit.

### Factors Associated With Dual Task Effects

Four participants did not undergo the neuropsychological test battery and could not be grouped according to cognitive status; they were therefore removed from this analysis. Further excluded from the analyses were those participants who were classified as being of intermediate cognitive status, that is, who could not with certainty be classified as either PD non-MCI or PD MCI (n = 24, 25.8% of total).

Results of the multivariate analyses are presented in Table 4. Cognitive status should be interpreted in such a way that an increase (ie, a positive value) represent the PD MCI group. A negative value on the DT variable is a DT cost, whereas a positive value is a DT benefit. All 3 models reached statistical significance (*P* < .05). A total of 20.9% of variance in DT effect on gait speed was associated with cognitive status and number of seconds to complete TUG-cog. These factors could also explain a total 15.7% of the variance in DT effect on step time variability. Last, 10.8% of variance in DT effect on cognition could be explained by independent variables cognitive status and MDS UPDRS part I.

**Table 4.** Multivariate Analysis Between Dual Task Variables and Independent Variables.

Dependent variable	Independent variable, unstandardized B (95% CI)	Adjusted *R*^2^
Dual task effect on gait speed (%)	Cognitive status: −7.408 (−12.662 to −2.155), *P* = .006 TUG-cog: −0.464 (−0.777 to −0.152), *P* = .004	0.209
Dual task effect on step time variability (%)	Cognitive status: −28.427 (−48.967 to −7.888), *P* = .007 TUG-cog: −1.330 (−2.552 to −0.107), *P* = .034	0.157
Dual task effect on cognition (%)	Cognitive status: 5.711 (0.652 to 10.770), *P* = .028 MDS-UPDRS I: −0.654 (−1.163 to −0.146), *P* = .013	0.108

Abbreviations: CI, confidence interval; TUG-cog, Timed Up and Go Cognitive; MDS-UPDRS, Movement Disorder Society–Unified Parkinson’s Disease Rating Scale.

### Dual Task Effects, Prioritization, and Cognitive Status

Participants in the PD MCI group exhibited a significantly larger DT cost on gait speed (mean −9.1%, SD 12.9) compared to the participants in the PD non-MCI group (mean −1.0%, SD 9.2), mean difference 8.1% (95% CI = 13.6 to 2.6; *P* = .005), which remained statistically significant when adjusted for age and sex (7.8%, 95% CI = 13.7 to 2.0; *P* = .009). Both groups also experienced DT cost on step time variability, but it was significantly higher in the PD MCI group (mean −41.8%, SD 49.9) compared to PD non-MCI participants (mean −11.4%, SD 33.8). There was a mean difference of 30.4% (95% CI = 51.3 to 9.5; *P* = .005), which also remained significant when adjusted for age and sex (32.6%, 95% CI = 54.7 to 10.4; *P* = .005).

Dual task costs on cognition were −7.2% (SD 8.0) and −3.0% (SD 12.5) in the PD non-MCI group and PD MCI group, respectively, but this across group difference was not significant (mean difference 4.2%; 95% CI = −1.0 to 9.3; *P* = .108).

The PD non-MCI group prioritized gait speed over performance on the cognitive task (*P* = .003), while the PD MCI group, on the other hand, prioritized cognitive task performance over gait speed (*P* = .044). The across-group effect of prioritization between cognition and gait speed was significant, with a mean difference of 15.6 (95% CI = 7.2 to 24.1; *P* < .001), and these results remained significant when adjusted for age and sex (15.3, 95% CI = 6.2 to 24.5; *P* = .001).

**Figure 2.**
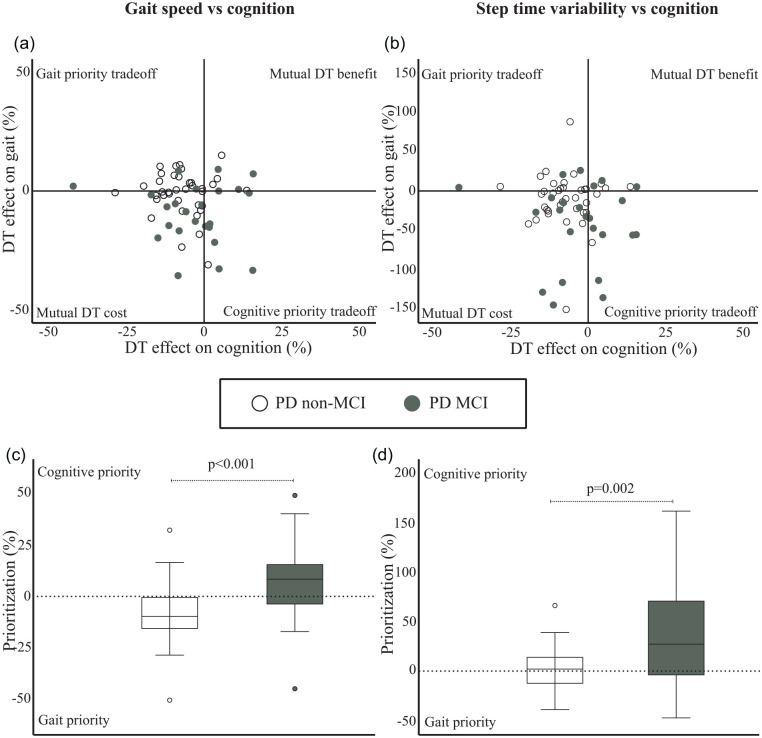
Patterns of individual dual task (DT) effects between cognition and gait speed (2a), and step time variability (2b), as well as prioritization shown as boxplots (median, interquartile range and outliers) between the cognitive task and gait speed (2c), and step time variability (2d) in the PD non-MCI and PD MCI groups respectively.

The PD MCI group also had a significant prioritization of cognitive task performance over step time variability (*P* = .001), whereas the mean prioritization values for the PD non-MCI was not significantly different from zero (*P* = .587) indicating a lack of prioritization of one task over the other. There was an across-group effect with significantly higher positive values in the PD MCI group than the PD non-MCI group, mean difference 39.3 (95% CI = 18.5 to 60.0; *P* < .001) and this remained statistically significant after adjusting for age and sex (39.4, 95% CI = 17.4 to 61.3; *P* = .001). Figure 2a to d presents these patterns of DT effect and prioritization, respectively, for the PD non-MCI and PD MCI groups.

## Discussion

In this sample of people with mild to moderate PD, DT costs were observed across all gait domains except asymmetry. There was a DT cost on cognition as shown by increased RTs and intraindividual variability on RT, but accuracy remained similar from single to DT performance. Cognitive status and TUG-cog were independently associated with DT cost on gait speed, step time variability, and cognition. Apart from cognitive status and TUG-cog, nonmotor experiences of daily living (part I of the MDS-UPDRS) was also associated with DT cost on cognition. People with PD MCI had larger DT cost on both gait speed and step time variability compared to PD non-MCI, but there was no across-group difference in DT cost on cognition. The PD MCI group consistently prioritized the cognitive task over gait, both regarding speed and step time variability. The PD non-MCI group instead prioritized gait speed over cognition, whereas they did not prioritize one task over the other with respect to cognition and step time variability.

The general finding of DT costs within 4 out of 5 domains of gait is supported by other studies reporting impaired performance during dual tasking within the pace,^14,16,44-51^ rhythm,^14,47,48,50,51^ variability,^14,45,48,49^ and postural control domains.^48^ In the current study, DT costs in the variability domains were not only significant across all variables, but these costs were also the highest compared to the other domains. This may be related to variability being particularly vulnerable to the shift from automatic to consciously controlled gait.^52^ This has also been highlighted by studies in both healthy people,^53^ and people with PD,^54^ showing that despite instructions during DT paradigms to concentrate on “consistent” or “safe” walking, variability measures remain unchanged or even worsen. The lack of a DT cost on gait asymmetry in our study is in line with some studies,^55-57^ but contradicts others.^47,58^ Given the laterality of onset symptoms, gait asymmetry may be more prominent in the earlier stages of the disease.^18^ Since we excluded people with only unilateral involvement (ie, Hoehn & Yahr 1), this may hypothetically explain why no significant DT effects on asymmetry could be found.

The relative change in performance from single to DT, that is, the DT effect, represents the interaction between the 2 tasks.^59^ Identifying factors associated with DT effect during walking has however turned out to be challenging. Our results show that both motor and cognitive function can explain small parts of the variance, but a large proportion remains unaccounted for, mirroring the results of previous studies.^5,16^ We found that cognitive status could explain parts of the variance in DT effect in all 3 models. In contrast to our hypothesis motor function, as assessed with MDS-UPDRS III, was not associated with DT effect. Instead, more severe nonmotor symptoms of daily living (MDS-UPDRS I; which assesses cognitive ability, mood, pain, and others^20^) were associated with increased DT cost on cognition. Interestingly, other more comprehensive scales related to depression, anxiety, health status, or quality of life did not correlate highly enough with DT effect variables to be included in the models. There are however differences in both scope and wording between MDS-UPDRS part I, which convey the extent to which symptoms affect performance of activities of everyday life, and scales like PDQ -39 and HADS that instead concern how often various types of symptoms occur. More research is needed to elucidate the large, unexplained part of the interaction between single and DT performance, and how it associates not only to motor and cognitive abilities, but also to self-rated symptoms.

Interestingly our sample of PD non-MCI participants prioritized gait speed over the cognitive task (posture-first strategy), whereas the PD MCI participants used a posture-second strategy regarding both gait speed and step time variability. This both confirms and contradicts previous findings. Bloem et al reported that a sample of PwPD without cognitive impairment used a posture-second strategy,^7^ whereas Yogev-Seligmann et al instead showed that PwPD without cognitive impairment used a posture-first strategy, and especially when instructed to do so.^8^ Our sample was not instructed to prioritize one task over the other and therefore those with PD non-MCI seemed to use this posture-first strategy instinctively. As has been suggested previously unconscious prioritization during DT walking can most likely not be explained by one sole determinant. It may primarily relate to motor and cognitive reserve, but also to personality, expertise, and affect state.^60^ Our between-group analyses on prioritization was controlled for sex and age only and does not allow for consideration of other possible factors. Given that certain neuropsychiatric symptoms, such as depression and apathy, are more prevalent in PD MCI than PD non-MCI,^61^ it is reasonable to think that such differences could have played a role in the prioritization pattern of our groups.

To our knowledge, this is the first study to use a level II criteria for MCI classification in elucidating the association with DT costs on gait, and differences in patterns of cognitive-motor interference according to cognitive status in PD. Other studies with similar focus have used a less comprehensive neuropsychological test battery for classification^14,15^; however, it is unclear to what extent this may affect the validity of comparing our results to theirs. Gassner et al found no group differences in DT costs between their sample of PD MCI and PD non-MCI participants,^14^ whereas Amboni et al found a difference between PD MCI and PD non-MCI only in the single/double support time ratio, and only during OFF medication.^15^ Neither of these reported performance of the cognitive task, and so it is however possible that a DT cost existed there. A recent study showed that in their sample of people with PD who scored high (≥26) on MoCA, a high percentage (45%) actually exhibited cognitive decline on 2 or more neuropsychological tests.^62^ This complexity in identifying cognitive impairment is perhaps also reflected in the heterogeneous results of studies on DT gait in PD.

This study has several strengths and limitations. Our relatively large sample (N = 93) was comprehensively assessed both in relation to gait and cognitive abilities. We further collected an extensive amount of self-reported as well as clinically assessed descriptive data which allowed many potential confounders to be controlled for. No power calculation was however conducted for these specific aims since it was based on baseline data from a randomized controlled trial. Some aspects relating to the main outcome variables in this study also warrant attention. The DT effect on gait speed while performing auditory Stroop has shown moderate test-retest reliability (ICC 0.61),^34^ whereas to our knowledge no reliability assessments have been reported previously for DT effect variables on step time variability in PD. We also chose to calculate this overall DT effect on cognition in order to consider both RT and accuracy, as previously presented in supplementary material by Strouwen et al.^63^ Our intention was to ensure that a low cost on RT was not simply derived from an increased cost on accuracy and vice versa. However, the reliability and validity of this variable needs further investigation. Moreover, 2 matters relating to the generalizability of the results also deserve attention. First, straight walking in a controlled laboratory setting without distractions from, for example, surrounding traffic or pedestrians has low ecological validity. Second, testing was conducted during the ON stage of the medication cycle, and results may therefore not transfer to when people are in the OFF stage of medication.

The results of this study highlight the need to extend DT gait analysis in PD to more parameters than speed. Also, given the preliminary evidence that people with and without PD MCI use different patterns of task prioritization this may call for different treatment approaches and different strategies when instructing people with PD in gait assessment and training situations. This finding however requires further investigation, and we recommend that future research into the role of cognitive status uses a comprehensive cognitive assessment battery instead of single global measures of cognition.

In conclusion, in our sample of people with mild to moderate PD, a DT cost during walking was exhibited across all gait domains except asymmetry. Although participants maintained high accuracy on the cognitive task, walking simultaneously significantly increased their RTs and intraindividual variability. We showed that prioritization differed according to cognitive status, whereby PD MCI used a posture-second strategy, and PD non-MCI used a posture-first strategy. Our findings provide evidence which can inform the planning and instruction of motor cognitive DT training and assessment, in accordance with cognitive status, in order to bring about more precise effects among heterogeneous groups of people with PD.
